# Aerobic and anaerobic iron oxidizers together drive denitrification and carbon cycling at marine iron-rich hydrothermal vents

**DOI:** 10.1038/s41396-020-00849-y

**Published:** 2020-12-17

**Authors:** Sean M. McAllister, Rebecca Vandzura, Jessica L. Keffer, Shawn W. Polson, Clara S. Chan

**Affiliations:** 1 School of Marine Science and Policy, University of Delaware, Newark, DE USA; 2 Department of Earth Sciences, University of Delaware, Newark, DE USA; 3 Center for Bioinformatics and Computational Biology, University of Delaware, Newark, DE USA; 4 Present Address: The Cooperative Institute for Climate, Ocean, and Ecosystem Studies, University of Washington, Seattle, WA USA; 5 Present Address: Pacific Marine Environmental Laboratory, National Oceanic and Atmospheric Administration, Seattle, WA USA

**Keywords:** Biogeochemistry, Environmental microbiology, Metagenomics, Microbial ecology, Microbial ecology

## Abstract

In principle, iron oxidation can fuel significant primary productivity and nutrient cycling in dark environments such as the deep sea. However, we have an extremely limited understanding of the ecology of iron-based ecosystems, and thus the linkages between iron oxidation, carbon cycling, and nitrate reduction. Here we investigate iron microbial mats from hydrothermal vents at Lōʻihi Seamount, Hawaiʻi, using genome-resolved metagenomics and metatranscriptomics to reconstruct potential microbial roles and interactions. Our results show that the aerobic iron-oxidizing Zetaproteobacteria are the primary producers, concentrated at the oxic mat surface. Their fixed carbon supports heterotrophs deeper in the mat, notably the second most abundant organism, *Candidatus Ferristratum* sp. (uncultivated gen. nov.) from the uncharacterized DTB120 phylum. *Candidatus Ferristratum* sp., described using nine high-quality metagenome-assembled genomes with similar distributions of genes, expressed nitrate reduction genes *narGH* and the iron oxidation gene *cyc2* in situ and in response to Fe(II) in a shipboard incubation, suggesting it is an anaerobic nitrate-reducing iron oxidizer. *Candidatus Ferristratum* sp. lacks a full denitrification pathway, relying on Zetaproteobacteria to remove intermediates like nitrite. Thus, at Lōʻihi, anaerobic iron oxidizers coexist with and are dependent on aerobic iron oxidizers. In total, our work shows how key community members work together to connect iron oxidation with carbon and nitrogen cycling, thus driving the biogeochemistry of exported fluids.

## Introduction

Chemolithotrophy fuels primary production and nutrient cycling in dark environments (e.g., [1–3]). This has been well demonstrated for deep sea sulfur-oxidizing ecosystems (e.g., [4, 5]), yet at the bottom of the ocean, the most abundant source of energy for chemolithotrophy is iron that originates from basaltic ocean crust [6]. Iron-oxidizing microbial communities can be found associated with widespread hydrothermal vents at the ocean floor (i.e., [7–17]). The best-studied example is Lōʻihi Seamount (also in publication as Loihi Seamount), a submarine volcano near Hawaiʻi with extensive iron microbial mats associated with low- to mid-temperature vents [11, 14, 18–20]. These distinctive biomineral mats at Lōʻihi are produced by the Zetaproteobacteria [21, 22], a class of aerobic autotrophic iron oxidizers that are the only known iron oxidizers in the mat. While the Zetaproteobacteria are relatively well-studied [7, 23, 24], the ecology of their microbial mats is poorly explored. Metabolic predictions are largely based on isolate physiology studies [25, 26] and genomic potential [19, 20, 24] of the Zetaproteobacteria, while the functions of other, flanking members of the microbial community have largely been inferred from 16S rRNA gene taxonomy, which assumes metabolism is tied with taxonomic affiliation [8, 11, 18]. However, this approach overlooks the roles of uncharacterized taxa such as the DTB120 phylum, found at Lōʻihi [11, 20], as well as viral communities that may moderate mat ecology and mediate nutrient fluxes [27]. Thus, major questions remain about the metabolisms and biogeochemical roles of these iron oxidation-driven ecosystems.

One key question is how iron oxidation drives carbon cycling throughout the iron mat. Lōʻihi mats are somewhat enriched in ^13^C [28], consistent with primary productivity. A study using quantitative PCR showed that the Calvin-Benson-Bassham (CBB) pathway gene *cbbM/rbcL* is much more abundant than *aclB* (reductive tricarboxylic acid pathway), suggesting that the CBB pathway is the dominant carbon fixation pathway in the mats [29]. However, not all carbon fixation pathways were investigated and the responsible organisms were not identified. Zetaproteobacteria are often abundant in the mats (ranging from 1 to 96%) [19, 24], and all have CBB pathway genes, based on isolate and environmental genomes [19, 20, 24–26, 30–33]. Thus, due to abundance and genetic potential, Zetaproteobacteria are the presumed primary producers in the Lōʻihi iron mats, yet this has not been definitively shown. Particularly since Zetaproteobacteria are aerobic and oxygen is typically depleted within the first few mm’s to cm’s of the mat surface [21, 34], there are large anoxic portions of the iron mat where we do not know the source of fixed carbon or the trophic structure of the community. To understand how carbon flow structures the ecosystem, we need to determine how both aerobic and anaerobic organisms contribute to carbon cycling.

Compared to carbon, we know even less about nitrogen cycling in iron-rich mats and vents. The main nitrogen sources at Lōʻihi are vent fluids containing ammonia (0.28–7.5 µM) and the surrounding ocean water containing nitrate (36–43 µM) [35–37]. Sylvan et al. [35] showed that Lōʻihi vent fluids have elevated nitrate N and O isotope ratios, with patterns that suggest denitrification combined with nitrification and/or ammonia oxidation. Some Zetaproteobacteria have genes for nitrate assimilation (*nasA*) and denitrification (*napA*/*nirK/nirS*) [7, 19, 29], but it is unclear if Zetaproteobacteria are the primary drivers of denitrification in the mats. Denitrification within the anaerobic portions of the mat is expected, though it is unknown whether this denitrification is coupled to the oxidation of organic carbon, Fe(II), or both. The presence of both nitrate and Fe(II) at Lōʻihi suggests there is a niche for nitrate-reducing iron oxidizers. However, there has been much debate about whether denitrifying organisms can enzymatically conserve energy from iron oxidation as opposed to chemodenitrification, in which organotrophic denitrification produces nitrite that oxidizes Fe(II) (see review by Bryce et al. [38]). While nitrate reducers that clearly enzymatically oxidize iron largely elude isolation, a number of studies have demonstrated the relevance of coupled iron oxidation and denitrification in coastal marine environments [39, 40], suggesting that it may also be important in other marine environments. These issues are important to resolve if we are to understand how iron, carbon, and nitrogen cycling are linked in deep sea iron systems.

To reconstruct microbial interactions that connect iron oxidation with nutrient cycling, we conducted a genome-resolved metagenomics and metatranscriptomics study at Lōʻihi Seamount. We aimed to better understand the balance of metabolic processes and contributions of specific organisms throughout the mat, including organisms like viruses that have not otherwise been surveyed in iron mats. We collected a surficial mat sample to represent aerobic processes, as well as two bulk samples, which include deeper, anaerobic portions of the mats. The surface sample and one bulk sample were preserved in situ for metatranscriptome studies. We used the other bulk sample in a shipboard incubation experiment in which we added Fe(II) and oxygen to stimulate aerobic iron oxidation and monitored the transcriptomic response of the community. Our results reveal a fuller picture of the microbial ecology and geochemical cycling in iron microbial mats, including viral influences on dominant community members, nitrogen cycling by Zetaproteobacteria, and a role for a potential nitrate-reducing iron-oxidizing *Candidatus Ferristratum* sp., from the uncharacterized DTB120 phylum.

## Methods

A complete description of sample collection, the Fe(II) amendment experiment, DNA and RNA extraction and sequencing, metagenomic analysis, and RNA read recruitment are provided in McAllister et al. [24], including detailed supplemental information with sample metadata. Here we briefly summarize these methods for Lōʻihi Seamount and describe additional data analysis for this manuscript.

### Sample collection

Three samples were collected at Lōʻihi Seamount, Hawaiʻi, in March 2013, using the remotely-operated vehicle (ROV) Jason II on the research vessel Thomas G. Thompson. One sample was collected via syringe sampler (~10 mL), collecting only the top cm of iron mat (S1). The other two samples were collected in bulk, with S19 by scoop sampler (~2 L) and S6 by suction sampler (>5 L). To preserve in situ RNA expression profiles, S1 was collected in a syringe half-filled with 2X RNALater (Invitrogen, Carlsbad, CA, USA) and S19 was collected in a two-chamber scoop where mat material collected in the first chamber was mixed with 2X RNALater from the second chamber immediately after sampling. Samples were allowed to settle for a few hours at 4 °C to concentrate the mat before being frozen at −80 °C.

### Fe(II) amendment experiment

A shipboard Fe(II) amendment experiment was performed on the bulk S6 sample, which included iron mat and entrained seawater. Briefly, mat material collected and retrieved after 2 h of ROV operations was allowed to settle at 4 °C for 1 h. Two serum bottles were filled with 250 mL of iron mat floc, with one bottle treated with 1 mM sodium azide 5 min prior to the start of the experiment, for a killed control. For the duration of the experiment, both bottles were mixed thoroughly by hand in a 35 °C water bath. A sample was taken 2 min prior to Fe(II) amendment (labeled pre-Fe(II) addition). To initiate the experiment, 100 µM FeCl_2_ was added to both bottles. After this addition, at 10 min intervals for 40 min, 30 mL of sample was removed and mixed 1:1 with 2X RNALater. Samples were stored at 4 °C for a few hours prior to freezing at −80 °C.

### DNA and RNA extraction and sequencing

DNA was extracted from samples using the FastDNA SPIN kit for soil (MP Biomedicals, Santa Ana, CA, USA). RNA was extracted using the NucleoSpin RNA kit (Macherey-Nagel, Bethlehem, PA, USA). Both kit protocols were followed with modifications (see [24]). Microbial community composition was estimated using a 16S rRNA gene survey using long-read PacBio sequencing, with taxonomy assigned using SILVAngs [41]. Metagenome (MG) and metatranscriptome (MT) libraries were sequenced at the University of Delaware Sequencing and Genotyping Center on an Illumina HiSeq 2500 [24].

### Metagenome and metatranscriptome analysis methods

Quality-controlled metagenome sequences were assembled using metaSPAdes v3.10 [42] and binned into metagenome-assembled genomes (MAGs) using DAS Tool [43] to select the best non-overlapping bins from MaxBin [44], MetaBAT [45], CONCOCT [46], and BinSanity [47], followed by manual curation in ggkbase (https://ggkbase.berkeley.edu/) and Anvi’o [48]. Of the total 215 MAGs from this study, 49 belonged to the Zetaproteobacteria [24]. Quality-controlled metatranscriptomic reads were recruited to curated MAGs and unbinned contigs using BEDTools [49]. RNA expression estimates were calculated from raw read recruitment numbers by normalizing these numbers for sequencing depth and gene and read length using the transcripts per million (TPM) metric [50]. Sample S6 expression, unless otherwise noted, is represented as an average of the expression over the time series. MG and MT relative abundances were calculated as a percentage of reads mapping to binned contigs and contigs that were identified as viral (see below). Maximum normalized TPM values were calculated by dividing TPM by the maximum TPM value across the S6 time series. Testing differential expression in DESeq [51], based on overall gene expression and the expression of genes by MAGs, failed to detect any significantly differentially expressed genes. This is likely due to a lack of replication from sampling limitations, resulting in low statistical power.

### Gene identification

Curated MAGs were submitted to RAST to be annotated by the SEED database [52] and to GhostKoala to be annotated by the KEGG database [53]. Gene calls from RAST were used for all analysis. Genes of interest were identified primarily through HMM searches via the LithoGenie program (github.com/Arkadiy-Garber/LithoGenie). This program uses validated HMMs to identify genes in the C, Fe, N, S, As, and H cycles (Supplementary Table 1) (see supplemental data 14 in [54]), including the three clusters of the putative iron oxidase, *cyc2* [55]. The clustering of the *cyc2* genes was confirmed by phylogenetic reconstruction and comparison with a reference database [24]. A small subset of genes were identified using NCBI BLASTp [56].

### DTB120 Identification

To classify the unknown DTB120 from Lōʻihi, we selected 16S rRNA gene representatives from the Desulfobacterota (closest relative; previously known as the Deltaproteobacteria [57]), Zetaproteobacteria, Gallionellaceae, Aquificae, and Chloroflexi within the SILVA database [58]. A 16S rRNA phylogenetic tree of these reference sequences and all DTB120 sequences was constructed from a SINA alignment [59] using RAxML [60] and visualized with Iroki [61].

To identify DTB120 MAGs, we first identified DTB120 16S rRNA genes within all bins initially classified as Desulfobacterota (closest relatives in Phylosift classifier [62]) using the ssu_finder in CheckM [63]. Only four MAGs possessed partial 16S rRNA genes. From these reference genomes, we used average amino acid identity (AAI; see supplement) and average nucleotide identity (ANI; data not shown) to identify closely-related MAGs that also belonged to the DTB120 (github.com/mooreryan/aai). These MAGs were named *Candidatus Ferristratum* sp.

### Viral identification

Metagenomic contigs representing near-complete phage genomes or containing prophage regions were identified using VirSorter 1.0.3. [64] (lower confidence categories 3 and 6 excluded). Viral contigs were annotated using the VIROME pipeline [65]. Viral taxonomy was determined in two ways: (1) with vContact2 using BLASTp against the ProkViralRefSeq94-Merged database [66] by assigning consensus taxonomy to clusters requiring 100% reference agreement to assign taxon at a given rank; (2) through consensus taxonomy of Best BLASTp Hit (BBH; *p* < 1e−5) for each ORF, with taxon assignment at a rank requiring ≥2 BBH and >50% of all BBHs in agreement [67]. Host-virus interactions were determined using CASC [68]. CRISPR spacers were blasted (BLASTn) against our assembled metagenomes for matches of >98% similarity.

### Data accessibility

Raw 16S rRNA gene, metagenome, and metatranscriptome reads were deposited in the NCBI SRA under BioProject accession PRJNA555820. Metagenome assemblies and high-quality MAGs were submitted to the JGI IMG database under sequence project IDs Gp0295814-Gp0295816. All MAGs are available for download from doi:10.6084/m9.figshare.12986078.v1. Additional sample metadata are available in [24].

## Results

### Community composition and activity

**Fig. 1:**
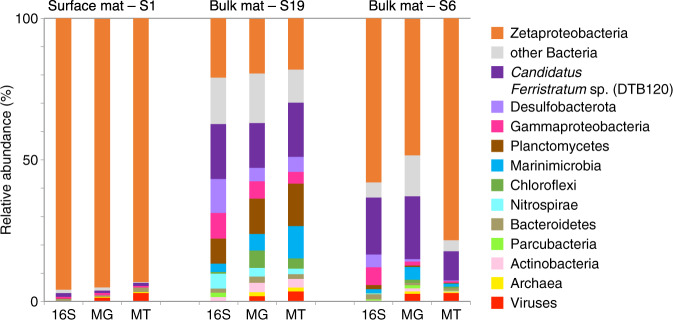
Lōʻihi Seamount iron mat microbial community composition based on 16S rRNA gene, metagenome (MG), and metatranscriptome (MT) relative abundance. Zetaproteobacteria and *Candidatus Ferristratum* sp. were the most abundant taxa. The 16S rRNA gene surveyed the bacterial population only. Viral expression based on viral contigs identified from VirSorter. S6 expression shown for pre-Fe(II) addition sample only.

Three samples from Lōʻihi Seamount iron mats were collected: in situ-preserved surface mat S1, in situ-preserved bulk mat S19, and shipboard-preserved bulk mat S6. We evaluated the community structure and activity of the iron mats at Lōʻihi Seamount (Fig. 1) using bacterial 16S rRNA amplicon libraries, metagenome (MG) recruitment, and metatranscriptome (MT) recruitment. We found the abundance estimates provided by all three measurements of microbial community composition were largely consistent with each other, suggesting that the active microbial populations are relatively stable in this iron mat environment.

The most abundant taxonomic group in the Lōʻihi iron mats was the Zetaproteobacteria, dominating the surface mat sample S1 at 95%/93% of the community (by MG abundance and MT activity, respectively) (Fig. 1). This high representation of Zetaproteobacteria at the surface of the actively growing mat is expected, due to their role in constructing the iron oxyhydroxide framework of the mat [21]. We were surprised to find the second most active population in the surface mat sample were viruses (3.1% by MT abundance). In the bulk mat samples S6 and S19, which include deeper mat, there was a lower abundance of Zetaproteobacteria, accounting for only 48%/19% of the S6/S19 community by MG abundance and 78%/18% by MT. Compared to the surface mat S1, the bulk mat samples were much more diverse, including (in order of decreasing abundance) DTB120, Desulfobacterota, Planctomycetes, Gammaproteobacteria, Marinimicrobia, and Chloroflexi. There was a minor population of Archaea in the bulk mat samples, and viral activity was detected in both bulk samples as well.

### Significant members of the flanking microbial community

#### *Candidatus Ferristratum*

The second most abundant group in the bulk samples was the DTB120 (0.7% by MG recruitment in the surface sample S1, 16% in S19, and 22% in S6, Fig. 1). DTB120 is an uncharacterized phylum, named for an uncultured clone from a hot spring microbial mat [69], with closest relatives in the Desulfobacterota. Sequences from the DTB120 had a median 16S rRNA sequence identity of 87.6% (minimum 79.0%). The closest cultured relative to our DTB120 sequences is *Syntrophorhabdus aromaticivorans*, a syntrophic, aromatic compound-degrading microbe isolated from anaerobic digesters [70, 71]. Based on a 16S rRNA gene phylogeny of DTB120 (Supplementary Figs. 1 and 2), *Syntrophorhabdus* spp. are at the base of the DTB120 phylum. However, DTB120 from our samples and other iron-rich hydrothermal vents and seeps (Lōʻihi Seamount, South Tonga Arc, Nile Deep Sea Fan) [11, 16, 20, 72] form a distant and distinct cluster within the DTB120 (minimum 90.1% identity, Supplementary Fig. 1), leaving the Lōʻihi DTB120 energy metabolism an open question.

Nineteen Lōʻihi DTB120 MAGs clustered into a single genus (62.0–98.6%, 77.0% average amino acid identity (AAI); above the 65% cutoff proposed in [73]) with four of those MAGs positively identified as DTB120 based on partial 16S rRNA genes (Supplementary Fig. 3). Clustering by average nucleotide identity (ANI) yielded an equivalent result (data not shown). Within this DTB120 genus, which we have named *Candidatus Ferristratum* sp., four subclusters of genomes shared a high enough similarity to form species with more than one genome representative (>95% AAI; Supplementary Fig. 3B; [73]). However, these species were only represented by a maximum of two MAGs, and were thus not considered to be sufficiently represented to warrant naming at a species level. Of the 19 recovered *Candidatus Ferristratum* sp. MAGs, 9 are sufficient in quality (<20% redundancy) and completeness (>70% complete) for analyses to predict the metabolic capabilities of the genus (see below; genomes marked with an asterisk in Supplementary Table 2), with the other 10 used for supporting information (Supplementary Table 2). Due to the similarity in their metabolic potential, *Candidatus Ferristratum* sp. MAGs are largely discussed as a unit.

#### Viruses

The iron mats all had substantial viral activity, which has not previously been characterized. To investigate the types of viruses and their potential hosts, we analyzed viral contigs and identified CRISPR spacers within MAGs (Supplementary Table 3). A total of 409 viral contigs (1–82 kb; median 4.7 kb) were identified. Based on consensus taxonomic placement, 85% of contigs were taxonomically associated with the tailed-phage order Caudovirales (Supplementary Fig. 4), with the family Siphoviridae the most numerous in all three samples (Supplementary Table 3). Among other viral groups, three contigs were placed in the ssDNA virus family Inoviridae and several contigs showed similarity to unclassified Halovirus. There were more taxonomically-unassigned contigs within the bulk samples (S19, S6) than in the surface (S1), which indicates an increased novelty of phage in the deeper mat layers.

Aside from several viral contigs that showed consistent Desulfobacterota and Gammaproteobacteria associations, most contigs indicated mixed class- or phylum-level hosts. While not uncommon to see lack of host specificity in a protein homology analysis, the significant variability here is likely indicative of under-characterized phage associations with the dominant microbes, Zetaproteobacteria and *Candidatus Ferristratum* sp. Host-association was also evidenced within MAGs, as many possessed CRISPR spacer arrays, including the Zetaproteobacteria and *Candidatus Ferristratum* sp. (Supplementary Table 4). We were able to match two CRISPR spacer sequences from Zetaproteobacteria MAG S1_Zeta3 with 100% identity to a 28-kb viral contig from S1 containing the nearly complete genome (43 ORFs) of a lysogenic Mu-like phage carrying a Zetaproteobacteria sulfate transporter. One of the spacers was in the first position, indicating the most recent infection, while the existence of a second spacer demonstrated repeated contact between the Zetaproteobacteria and the phage. Numerous other likely lysogenic phage are evident in the libraries including additional Mu-like phage, filamentous Inoviridae, and several identified prophage elements within the metagenomes (VirSorter categories 4 and 5 in Supplementary Table 3) consistent with past findings of lysogeny in association with microbial mats and effluent associated with geothermal features and other aquatic environments [74, 75]. Such repetitive contact and development of lysogeny indicate long term viral-host interaction, which may have implications for virally-mediated evolution through selective pressure, lysogenic conversion (alteration of host phenotype), and horizontal gene transfer [76].

### Lōʻihi mat community metabolisms

#### Carbon fixation

**Fig. 2:**
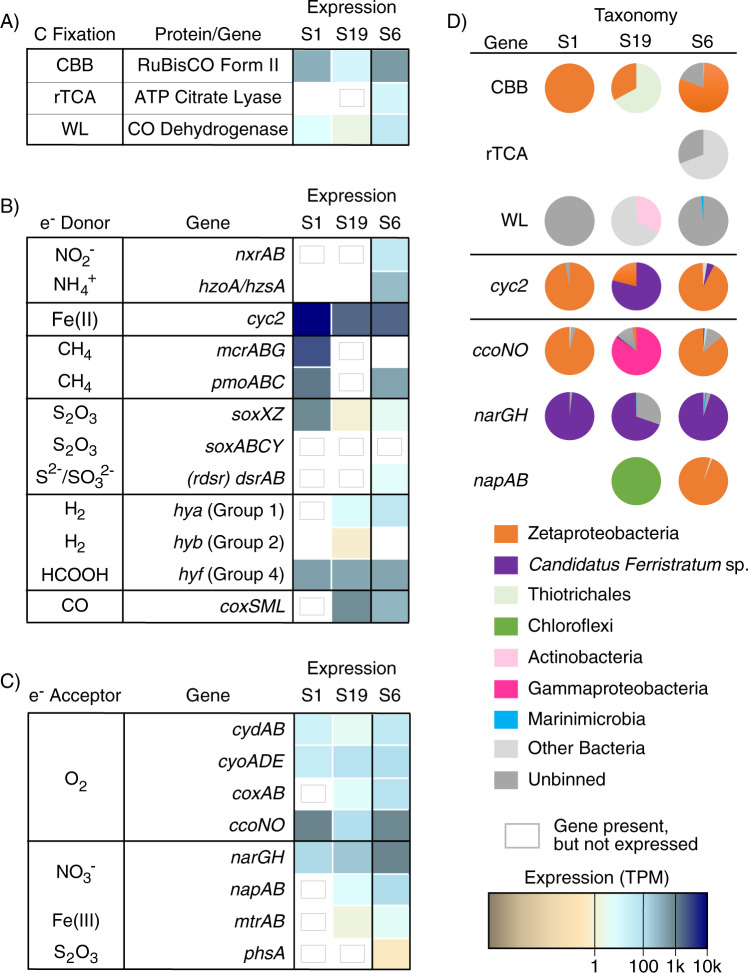
Gene expression for carbon fixation, electron donors and electron acceptors within the iron mat. Heatmaps show the total normalized gene expression (TPM) for **a** carbon fixation, **b** electron donors and **c** electron acceptors, with **d** pie charts showing the relative taxonomic contribution to expression. Genes that lacked expression that were still found within MAGs are indicated with gray boxes. Pathways that were not represented in a given sample remain white. Carbon fixation genes were expressed primarily by the Zetaproteobacteria (CBB), though other organisms were also involved: unbinned *Methanoperedens* sp. (WL) in S1, unbinned Nitrospirae (rTCA and WL) in S6, and Gammaproteobacteria Thiotrichales (CBB) and Actinobacteria (WL) in S19. In addition to iron oxidation, methane oxidation was another significant source of energy in S1, carried out by the unbinned Archaea *Methanoperedens* (*mcrABG*) and by the Gammaproteobacteria (*pmoABC*).

Zetaproteobacteria are thought to be the primary producers within iron mats, using the Calvin-Benson-Bassham (CBB) cycle [7, 30, 77, 78]. We tested this assumption using HMM profiles from five major carbon fixation pathways (Supplementary Table 1). RuBisCO Form II (catalytic subunit; *rbcL*) of the CBB pathway was the highest expressed carbon fixation gene in all three samples, at 53×, 17×, 5× higher than the next highest expressed carbon gene for S1, S6, and S19, respectively (Fig. 2a). Surface sample S1 and Fe(II)-amended bulk microcosm S6 had the highest expression levels of RuBisCO Form II, 27–50× higher than in the bulk mat S19, and the majority of those transcripts mapped to Zetaproteobacteria MAGs (Fig. 2d).

#### Heterotrophy

Our datasets provide the opportunity to make specific predictions about heterotrophic pathways and organisms. Many MAGs contained genes for degrading polysaccharides such as cellulose and chitin (Supplementary Table 5) [79]. The bulk mat sample S19 contained the greatest number of polysaccharide degradation genes, more than double the number found in surface mat S1 and microcosm S6. The *Candidatus Ferristratum* sp. MAGs all lacked autotrophy genes, but several possessed and expressed genes used for polysaccharide degradation. Almost all *Candidatus Ferristratum* sp. MAGs possessed genes for mannose transport and mannose-6-phosphate isomerase, which channels mannose into glycolysis (Supplementary Table 6). Thus, our analyses uncovered specific evidence for heterotrophy in *Candidatus Ferristratum* sp. and other flanking community organisms from Lōʻihi iron mats.

#### Electron donors

**Fig. 3:**
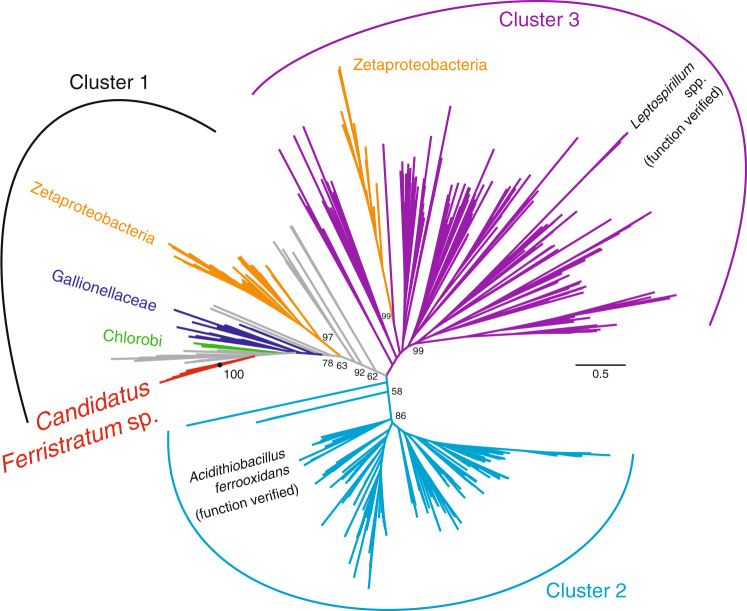
Cyc2 maximum likelihood phylogenetic tree (300 bootstraps) showing the relative placement of Cyc2 belonging to *Candidatus Ferristratum* sp. MAGs identified in this study. The *cyc2* genes found within the *Candidatus Ferristratum* sp. MAGs form a monophyletic clade (100% bootstrap support) distinct from the Zetaproteobacteria.

All three Lōʻihi iron mat samples have high expression of *cyc2*, a potential marker gene for Fe(II) oxidation (Fig. 2b) [24]. This gene is expressed in all three samples, 2.4–5.3× higher than genes for any other energy metabolism (Fig. 2b), making iron oxidation the key metabolism in the iron mats. The *cyc2* gene is most highly expressed in the surface mat S1, which is almost entirely composed of Zetaproteobacteria, and indeed, Zetaproteobacteria account for nearly all the *cyc2* expression in S1 (Fig. 2d). Zetaproteobacteria also dominate *cyc2* expression in the Fe(II)-amended bulk microcosm S6, with a small proportion of expression from *Candidatus Ferristratum* sp. In contrast, the high level of *cyc2* expression in the bulk mat S19 can be attributed primarily to *Candidatus Ferristratum* sp. While the high *cyc2* expression by Zetaproteobacteria is consistent with previous work [23, 24], *cyc2* expression by *Candidatus Ferristratum* sp. was an unexpected novel finding, as Zetaproteobacteria were previously the only hydrothermal iron mat organisms known to possess the *cyc2* gene. Phylogenetic reconstruction of Cyc2 sequences from public databases and our samples show that the *Candidatus Ferristratum* sp. Cyc2 sequences form a monophyletic clade within Cluster 1 (Fig. 3). Their closest neighbors are the Gallionellaceae and Chlorobi, suggesting that the *Candidatus Ferristratum* sp. did not acquire *cyc2* directly from the Zetaproteobacteria. Because Cluster 1 is largely comprised of Cyc2 from established neutrophilic iron-oxidizers, this presents the possibility that the *Candidatus Ferristratum* sp. may also be an iron oxidizer.

While Fe(II) oxidation appears to be the predominant energy acquisition pathway in all three Lōʻihi mat samples, genes for utilization of a diversity of electron donors are expressed by the non-Zetaproteobacteria flanking community, with notable differences between the surficial and bulk mats (Fig. 2b; taxonomic distribution for all genes in Supplementary Table 7). In the surface mat S1, there is high expression of genes for methane oxidation, consistent with previous reports of methane-oxidizers and methane oxidation genes within iron mats [72, 80, 81]. In contrast to the surface mat, bulk mat S19 and bulk microcosm S6 had higher expression of genes involved in H_2_ oxidation (Fig. 2b). Oxidative nitrogen transformations were also present but relatively rare in the iron mat genomes and metatranscriptomes (Fig. 2b).

#### Electron acceptors

Genes for oxygen and nitrate respiration were expressed in all Lōʻihi mat samples (Fig. 2c) with differences as expected due to the fact that S1 sampled the aerobic surface while S19 includes deeper regions of the mat [21, 34]. In the surface sample S1, the dominant terminal electron acceptor genes were the aerobic cbb_3_-type *ccoNO*, 11.8× more highly expressed than genes for the next electron acceptor, the respiratory nitrate reductase *narGH*, likely reflecting the greater availability of oxygen in the surface mat (Fig. 2c). Bulk mat sample S19 terminal electron acceptor expression was dominated by *Candidatus Ferristratum* sp. respiratory nitrate reductase *narGH* (Fig. 2d), though aerobic terminal oxidases were also expressed, primarily by Gammaproteobacteria. In addition, low levels of expression of nitrate reduction genes *napAB* and iron reduction genes *mtrAB* were detected. The bulk microcosm S6 expressed all terminal electron acceptors at the same or a higher level than bulk mat S19.

#### Nitrogen transformations

**Fig. 4:**
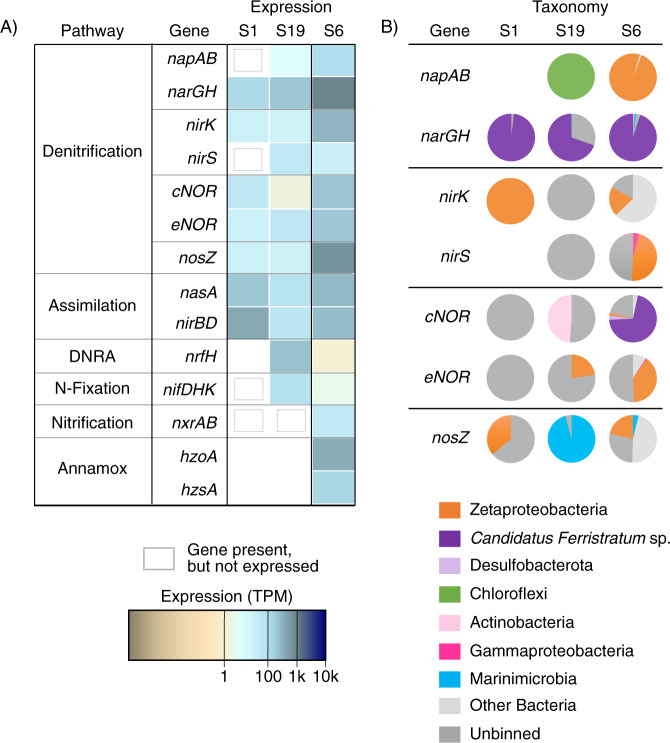
Nitrogen cycling gene expression within the iron mat. Heatmap shows the total normalized gene expression (TPM) for different metabolic pathways related to nitrogen cycling (**a**), with pie charts of relative taxonomic contribution to expression also shown (**b**). Genes that lacked expression that were still found within MAGs are indicated with gray boxes. Pathways that were not represented in a given sample remain white.

Previous work at Lōʻihi suggested that iron mat microbes actively oxidize and reduce nitrogen species [35]. To better understand the processes and taxa involved, we examined genes involved in nitrogen transformations (Fig. 4). Of these, genes for reductive nitrogen transformations were the highest expressed in all three samples. Genes for full denitrification of nitrate to N_2_ were expressed in all samples (Fig. 4a), including dissimilatory nitrate reductase (*napAB* or *narGH*), nitrite reductase (*nirK* or *nirS*), nitric oxide reductase (*norBC* or *eNOR* [a family of nitric oxide reductase with a putative proton channel, [82]]), and nitrous oxide reductase (*nosZ*)), but not all by the same organism (Fig. 4b). Instead, different parts of the pathway were expressed by *Candidatus Ferristratum* sp., Desulfobacterota, Zetaproteobacteria, Marinimicrobia, Sphingobacteriales, and other unclassified Bacteria.

Nitrogen fixation, nitrification, and anaerobic ammonia oxidation gene expression was detected in the bulk mat S19 and bulk microcosm S6 at low levels (Fig. 4a). Although *nirBD* plays a role in dissimilatory nitrate reduction to ammonium (DNRA), the majority of Zetaproteobacteria MAGs possess a gene cluster containing *nasA* and *nirBD* together, signaling these genes are likely used in concert for nitrate assimilation [7]. The expression of this assimilatory pathway was highest in S1 and S6, where the Zetaproteobacteria were most abundant. Assimilation of ammonium from environmental sources is mediated by the ammonium transporter encoded by *amt*, which is found in nearly all Zetaproteobacteria and 75% of the *Candidatus Ferristratum* sp. genomes.

### Fe(II)-amended bulk microcosm S6 time-series

To understand how iron oxidation affects community metabolisms, we added Fe(II) to the bulk mat sample S6 under aerobic conditions and then monitored gene expression every 10 min for 40 min, by which time Fe(II) was depleted. The S6 microcosm gene expression data showed a wide array of stimulated metabolisms, including both aerobic and anaerobic metabolisms and iron oxidation (Figs. 2b, c and 4a). In particular, Zetaproteobacteria *ccoNO* and *Candidatus Ferristratum* sp. *narGH* (Fig. 2c, d) were similarly highly expressed, and both taxa were actively expressing *cyc2* (Fig. 2c, d).

**Fig. 5:**
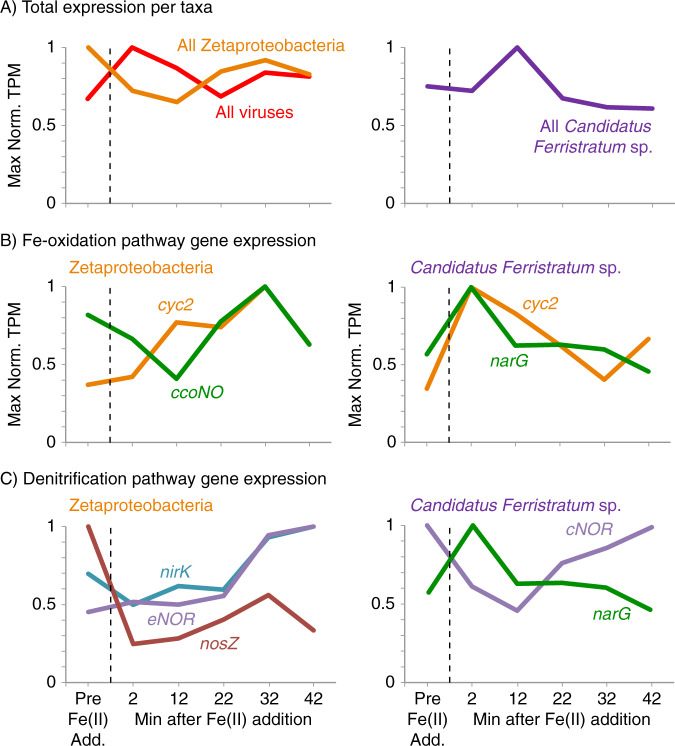
Gene expression patterns for major taxa during the S6 Fe(II) addition experiment. Maximum normalized gene expression (Max Norm. TPM) totals shown for organisms (**a**) and genes of interest (**b**, **c**) for the Zetaproteobacteria (left) and *Candidatus Ferristratum* sp. (right). Genes related to Fe(II) oxidation (*cyc2*, *ccoNO*, *narG*) (**b**) are separated from genes involved in denitrification (*narG*, *nirK*, *eNOR*, *cNOR*, *nosZ*) (**c**). Data are shown for the sample pre-Fe(II) addition, as well as at 10 min intervals starting 2 min after Fe(II) addition. Organism maximum TPM values: All Zetaproteobacteria (621,257), all *Candidatus Ferristratum* sp. (106,842), all viruses (37,024). Zetaproteobacteria gene maximum TPM values: *cyc2* (3,683), *ccoNO* (826), *nirK* (787), *eNOR* (31), *nosZ* (218). *Candidatus Ferristratum* sp. gene maximum TPM values: *cyc2* (110), *narG* (1,330), *cNOR* (106).

We evaluated how Zetaproteobacteria and *Candidatus Ferristratum* sp. transcription responded to Fe(II) stimulus by examining their individual gene expression patterns over the course of the time series (Fig. 5; we note that shipboard conditions precluded replicates and related statistical analyses). Immediately after Fe(II) addition, viral activity jumped, while Zetaproteobacteria decreased until 12 min (T12) before increasing. *Candidatus Ferristratum* sp. peaked at 12 min, suggesting *Candidatus Ferristratum* sp. and Zetaproteobacteria activity relate to different conditions (Fig. 5a).

To explore this, we examined genes potentially involved in the aerobic or anaerobic iron oxidation pathway (*cyc2*, *ccoNO*, *narG*) for each taxon (Fig. 5b). In the Zetaproteobacteria, total *cyc2* expression continuously increased after Fe(II) addition until 32 min (2.7× increase), in contrast to expression of the aerobic terminal electron acceptor (2.5× increase between lowest and highest values; Fig. 5b, left), which more closely followed the overall expression pattern of the dominant Zetaproteobacteria (Fig. 5a, left). This suggests that Zetaproteobacteria *cyc2* expression is stimulated by Fe(II) addition [24]. On the other hand, both *cyc2* and *narG* expression rapidly increased in *Candidatus Ferristratum* sp. within 2 min after Fe(II) addition (2.9× and 1.8×, respectively), followed by a decline (Fig. 5b, right). The *cyc2* and *narG* peak occurred before overall expression in *Candidatus Ferristratum* sp. peaked, suggesting these genes were specifically upregulated by Fe(II) addition before other cellular processes were stimulated by iron oxidation (Fig. 5a, b, right).

We also examined gene expression patterns for the denitrification pathway (*narG*, *nirK*, *eNOR*, *cNOR*, *nosZ*). With the exception of *narG*, the expression pattern shows some similarity between both groups of organisms, in some cases starting high and declining after Fe(II) addition, and then increasing after 2 or 12 min (Fig. 5c). This suggests that the mat already had sufficient capacity for transforming denitrification intermediates, such as nitrite, and new expression was not immediately necessary. Overall, the trends of Zetaproteobacteria and *Candidatus Ferristratum* sp. gene expression in response to Fe(II) addition suggest different niches and biogeochemical roles for these organisms within the gradients of iron mats.

### Contribution of individual Zetaproteobacteria and *Candidatus Ferristratum* sp. MAGs to iron and nitrogen mat cycling

**Fig. 6:**
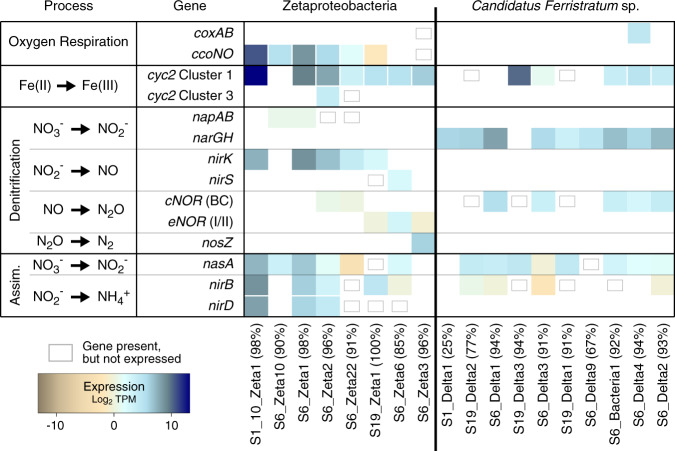
Heatmap showing the log_2_ total normalized gene expression (TPM) for different metabolic pathways related to iron, nitrogen, and oxygen cycling. Expression is shown for representative Zetaproteobacteria MAGs (left) and near-complete *Candidatus Ferristratum* sp. MAGs from S6/S19 and the only *Candidatus Ferristratum* sp. MAG from S1 (right). MAG percent completeness indicated in parentheses. Genes that lacked expression that were still found within MAGs are indicated with gray boxes. Pathways that were not represented in a given MAG remain white.

To better resolve the ecological niches of the major iron mat taxa, we examined the distribution and MAG-specific expression of genes within individual genomes, focusing on near-complete MAGs from the Zetaproteobacteria and *Candidatus Ferristratum* sp. (Fig. 6; *Candidatus Ferristratum* sp. details Supplementary Table 6). The majority of Zetaproteobacteria MAGs expressed both *cyc2* and *ccoNO* at high levels, as expected for aerobic iron-oxidizers (Fig. 6) [24]. Most of these MAGs also expressed the assimilatory nitrate reduction cassette with *nasA* and *nirBD*. Most MAGs expressed *nirK*, but the rest of the dissimilatory denitrification pathway was scattered between individual Zetaproteobacteria MAGs. Notably, several Zetaproteobacteria had the *napAB* nitrate reduction genes, though only two MAGs showed expression at low levels, compared to their aerobic respiratory *ccoNO* genes.

In contrast, *Candidatus Ferristratum* sp. metabolic potential was consistent between MAGs. Most of the MAGs possessed *cyc2*, though individual expression levels varied from undetectable to very high. Only one *Candidatus Ferristratum* sp. MAG possessed terminal oxidase genes (*coxAB*), while the majority instead expressed the *narGH* nitrate reductase genes (Fig. 6). None of the *Candidatus Ferristratum* sp. MAGs contained the complete denitrification pathway, only possessing *cNOR* nitric oxide reduction genes. Although *Candidatus Ferristratum* sp. appears to be anaerobic, several oxygen detoxification genes were found within the MAGs and were frequently highly expressed (Supplementary Table 6). Additionally, all high-quality *Candidatus Ferristratum* sp. MAGs possessed the oxygen-independent class II ribonucleotide reductase (RNR), and all but one also possessed the oxygen-sensitive class III RNR. These genes suggest that most *Candidatus Ferristratum* sp. are adapted to survive aerobic conditions. Given the high expression of *narG*, this suggests that the *Candidatus Ferristratum* sp. are primarily facultative anaerobes that respire nitrate and may be capable of iron oxidation.

## Discussion

Iron-oxidizing microorganisms can strongly influence the biogeochemical cycles of a wide range of elements. Previous studies have focused primarily on the biomineral byproducts, iron oxyhydroxides, which adsorb and coprecipitate various elements (e.g., [33, 83–86]). However, iron oxidizer physiology and ecological interactions are just as likely to affect biogeochemical cycling. Here, we have explored these interactions within three iron mat samples from Lōʻihi Seamount, comparing community composition and expression in aerobic surface mat (S1) with bulk mat that includes anaerobic niches (S6 and S19). In the S6 bulk mat microcosm, we were able to observe which organisms and processes are most primed to respond to Fe(II). With these samples, we explored the capabilities of individual iron mat members and reconstructed ecological interactions and community impacts on iron, carbon, and nitrogen cycles.

**Fig. 7:**
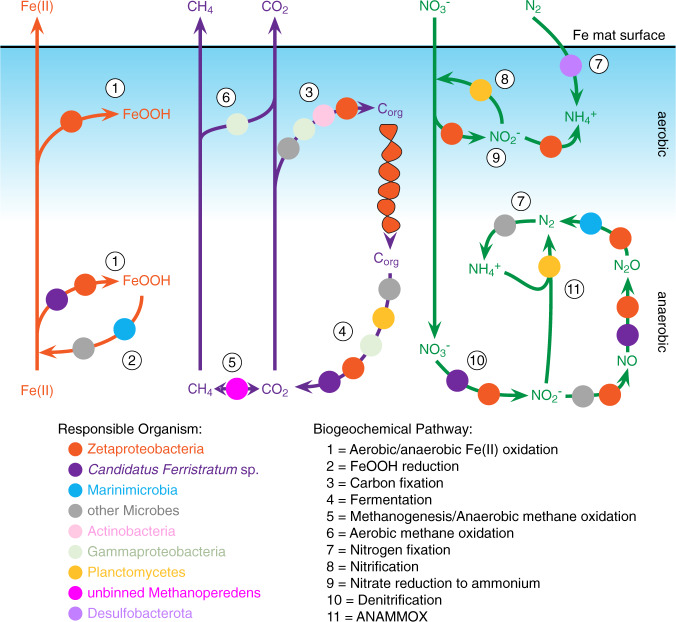
Cartoon showing the contribution of different members of the microbial community to iron, carbon, and nitrogen cycling within the aerobic/anaerobic gradient of an iron mat. The Zetaproteobacteria influence nearly every metabolic process in the mat, and support/are supported by a diverse flanking community, including the anaerobic *Candidatus Ferristratum* sp. The mat community is also supported by other metabolisms, such as oxidation of methane and H_2_.

The Lōʻihi Seamount iron mat communities are dominated by a few key players, primarily Bacteria, but also include Archaea and viruses (Figs. 1 and 7). The microbial community is structured by a gradient of oxygen, which becomes undetectable within the first few mm’s to cm’s of the mat surface [21, 34] Depending on oxygen levels, we find microorganisms involved in either aerobic iron oxidation (Zetaproteobacteria) or anaerobic iron oxidation (*Candidatus Ferristratum* sp.). These iron oxidizers (Fig. 7, process 1) form metabolic products (Fe(III), C_org_, NO_2_^−^) that support the metabolisms of flanking community microorganisms, thus either directly or indirectly driving most biogeochemical processes in the mat, including iron reduction, carbon fixation, fermentation, nitrate assimilation, and denitrification (Figs. 2 and 7).

Zetaproteobacteria are known to be autotrophs based on culture work and inference from MAGs [19, 20, 25, 77], but a definitive link to primary production in iron mats has not been demonstrated. Our work shows that the Zetaproteobacteria are the primary producers in the surface mat, where nearly all gene expression from known carbon fixation pathways can be attributed to RuBisCO Form II gene expression in the Zetaproteobacteria (Fig. 2). This suggests that carbon fixation is concentrated near the aerobic surface of actively growing mats. This is consistent with microscopy evidence that mats accrete as iron oxidizers form biomineral stalks, positioning the cells at the surface of the mat [21]. The iron oxyhydroxide stalk structures of the Zetaproteobacteria contain polysaccharides [87] and adsorbed organic exudates [88], thus the biominerals act as reservoirs of organic carbon for use by heterotrophs and fermenters. Viral induced lysis is another key mechanism for organic nutrient recycling, such as the viral shunt that maintains a pool of dissolved organic matter driving oceanic carbon cycling in the water column [89]. Given the diversity of phage, high abundance of viral transcripts in the metatranscriptomes, and evidence of repeated interaction between viruses and the dominant microbial populations in the mat, it is quite likely that viruses may be playing a similar role mediating carbon bioavailability in the iron mats (Supplementary Tables 3 and 4).

Zetaproteobacteria are aerobes, as no Zetaproteobacteria culture can grow anaerobically [7, 20] and all sequenced Zetaproteobacteria genomes include aerobic terminal oxidase genes [24]. However, some have the genetic potential for using nitrate reduction to live within an aerobic/anaerobic transition zone. A few Zetaproteobacteria genomes from the S6 bulk microcosm have the dissimilatory nitrate reductase genes *napAB* (Figs. 4 and 6). These organisms may conduct aerobic nitrate reduction, using nitrate as a backup electron acceptor under oxygen limiting conditions [90]. This is consistent with the concurrent expression of genes for terminal oxidase (*ccoNO*) and nitrate reduction (*napAB*) (Fig. 6) within the well-mixed S6 microcosm. Furthermore, different Zetaproteobacteria MAGs encode various other parts of the dissimilatory denitrification pathway (Fig. 6). Only the dissimilatory nitrate reductases are known to conserve energy in this pathway, though eNOR may also conserve energy via a proposed proton pump [82]. Thus, Zetaproteobacteria engaging in only part of the pathway may do so as a means of detoxifying intermediates, such as nitric oxide [91], or removing nitrite to avoid abiotic iron oxidation and encrustation in an Fe(II)-rich environment [38, 92]. Together with evidence of nitrate assimilation (including in [19, 20]), these results show that within the Zetaproteobacteria themselves, iron oxidation and nitrate reduction are coupled in multiple ways.

Since Zetaproteobacteria are primarily aerobes, this leaves anaerobic niches open for other organisms to thrive. *Candidatus Ferristratum* was overall the second most abundant taxa within the iron mats (Fig. 1) and their high expression of *narGH* (Figs. 2 and 4) and Cluster 1 Cyc2 homologs (Fig. 3) suggests they are active anaerobic denitrifiers with the ability to oxidize Fe(II). Given the rapid and parallel responses of *cyc2* and *narG* genes after Fe(II) stimulus (Fig. 5), *Candidatus Ferristratum* sp. may represent a novel nitrate-reducing iron-oxidizing taxon. Although oxygen was available in the microcosm, the injection of Fe(II) may have promoted transient anaerobic niches within the mat material. In the environment, *Candidatus Ferristratum* sp. are much more abundant in bulk mat samples (Fig. 1), including those from previous studies at Lōʻihi Seamount [11, 20]. Our findings suggest there may be two partitioned niches of iron oxidation in iron mats: The Zetaproteobacteria oxidizing iron in the shallow, aerobic zone, and *Candidatus Ferristratum* sp. conducting nitrate-reducing iron oxidation in the primarily anoxic zone.

**Fig. 8:**
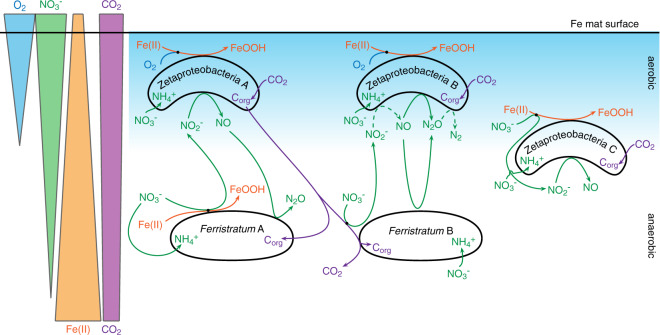
Cartoon model showing potential C and N cycling between the aerobic Zetaproteobacteria and anaerobic Candidatus Ferristratum sp. within the gradient of the iron mat. Letters denote either different Zetaproteobacteria taxa or different potential metabolic strategies in a single *Candidatus Ferristratum* sp. cell. Zetaproteobacteria A (ZA): Capable of Fe(II) oxidation using oxygen, assimilating nitrate, reducing nitrite, and fixing carbon. ZB: Capable of reducing nitrogen intermediates. All have NOR, though ones with NIR don’t have NOS. (see Fig. 6). ZC: A few Zetaproteobacteria with NapA may be able to couple nitrate reduction with iron oxidation (these Zetaproteobacteria also capable of the metabolism in ZA). *Candidatus Ferristratum* A: Capable of nitrate reduction coupled to iron oxidation. *Candidatus Ferristratum* B: Capable of nitrate reduction coupled to organic C oxidation.

The Lōʻihi iron mats should be an ideal place to find nitrate-reducing iron oxidizers, due to gradients of Fe(II), O_2,_ and NO_3_^-^ in the mat [21, 34, 35]. Nitrate reduction coupled to iron oxidation is theoretically possible for a single organism, but no isolate aside from the hyperthermophilic Archaea *Ferroglobus placidus* [93] has been shown to do so unequivocally [38, 94]. Indeed, the model system for nitrate-reducing iron oxidizing bacteria is the KS enrichment culture containing an autotrophic, nitrate-reducing iron-oxidizing Gallionellaceae partnered with heterotrophs that complement the Gallionellaceae’s denitrification pathway [94–96]. At Lōʻihi, Zetaproteobacteria produce organic carbon and consume oxygen, creating a niche for nitrate-reducing iron oxidizers. However, it is clear that Zetaproteobacteria themselves do not fill this niche, though they can assimilate nitrate using electrons from Fe(II) (Fig. 8, Zetaprotobacteria cell A), and some may conduct aerobic denitrification for redox balance (Zetaproteobacteria cell C). The anaerobic iron oxidation niche is open for another organism, such as the dominant denitrifier *Candidatus Ferristratum* sp., which may couple nitrate reduction to either iron (Fig. 8, *Candidatus Ferristratum* cell A) and/or organic carbon oxidation (*Candidatus Ferristratum* cell B). Either way, the high *Candidatus Ferristratum* sp. *narG* expression (Figs. 2 and 4) suggests that significant quantities of nitrite are produced in the mats. In theory, this could rapidly oxidize Fe(II) through chemodenitrification, which would result in the encrustation of nitrite-producing cells [38, 92]. However, Zetaproteobacteria expressed *nirK* highly, and increased *nirK* expression in the Fe(II)-amended bulk microcosm after peak *narG* expression (Fig. 5), suggesting that the Zetaproteobacteria actively remove nitrite produced by *Candidatus Ferristratum* sp. (Fig. 8, Zetaproteobacteria cell A). Zetaproteobacteria (cell B; Fig. 8) and *Candidatus Ferristratum* sp. (cell B) also express genes to reduce NO via eNOR and cNOR, respectively (Fig. 6), suggesting the need to detoxify NO. Finally, some Zetaproteobacteria can complete denitrification from N_2_O to N_2_ (cell B; Fig. 8). In this partner approach, a nitrate-reducing iron-oxidizing organism is relieved of the burden of having to synthesize all four separate enzyme complexes to denitrify to N_2_ because other taxa help with detoxifying byproducts. This cooperative approach to denitrification may explain why it has been challenging to isolate nitrate-reducing iron oxidizers.

## Conclusions and implications

At Lōʻihi Seamount, energy from iron oxidation fuels the growth and ecological interactions of a diverse microbial community. The well-known Zetaproteobacteria colonize iron-rich vents [18, 97], oxidize Fe(II) aerobically, and produce Fe(III) oxyhydroxide stalks to create the physical framework of the mat [21]. The Zetaproteobacteria use energy and electrons from Fe(II) to fix carbon, some of which binds to mat biominerals. Organic carbon is also made available through the viral lysis of Zetaproteobacteria, as they are hosts to Mu-like lysogenic phages and in contact with a diverse and active viral assemblage. Zetaproteobacteria oxygen consumption creates anaerobic zones and thus, in these various ways, Zetaproteobacteria create the physical and chemical niche for the nitrate-reducing heterotrophic iron oxidizer *Candidatus Ferristratum*. Because this metabolism is inferred from genomes and transcriptomes, the logical next step would be to attempt isolation. However, it is not clear that complete isolation would be successful, as *Candidatus Ferristratum* sp. appears to require others to remove byproducts to prevent chemodenitrification and toxicity. Instead, we can use our results as a starting point for more specific probing of ecological interactions and metabolite exchange within the mat, which may be unique in iron mats because of the affinity of organics for iron. In addition to affecting local biogeochemistry, organic-bound iron and other metabolites are carried by diffusely venting fluid moving through the mat, exported from Lōʻihi in buoyant plumes for 100 s to 1000+ km [98]. These exported fluids fertilize iron-depleted waters, connecting microbial iron and nutrient cycles across ocean basins.

### Description of *Candidatus Ferristratum* gen. nov

*Ferristratum* (fer.ri.stra’tum. L. neut. n. *ferrum* iron; L. neut. n. *stratum* mat/cover or a layer; N.L. neut. n. *Ferristratum* from an iron mat layer). Genus defined from nine metagenome-assembled genomes with >70% completeness and <20% redundancy with an average 77% pairwise AAI. Genome sources from three unique samples. Type material: MAG S6_Bacteria1 (partial 16S rRNA gene present). Five of the highest-quality (>90% complete, <5% redundant) *Candidatus Ferristratum* sp. genomes have been submitted to IMG under the analysis project IDs Ga0454285-Ga0454287, Ga0454293, and Ga0454316 (type material).

Physiological inferences from genome annotation: Heterotrophic. Facultative anaerobic. Capable of respiring nitrate coupled to organic carbon or Fe(II) oxidation for energy. Capable of nitric oxide reduction and nitrate assimilation. Found within Fe(II)-rich hydrothermal vent bulk/deep mat and sediment environments.

## Supplementary information

Supplemental Tables and Figures

Supplemental Table 1

Supplemental Table 3

Supplemental Table 6

Supplemental Table 7
