# Supplementary material for: Aerobic and anaerobic iron oxidizers together drive denitrification and carbon cycling at marine iron-rich hydrothermal vents
Source: ISME J. 2020 Dec 17;15(5):1271–86. doi: 10.1038/s41396-020-00849-y (PMC8114936; doi:10.1038/s41396-020-00849-y)
Supplement: Supplementary file 1 — Supplemental Tables and Figures [file 41396_2020_849_MOESM1_ESM.pdf]

Supplemental Tables and Figures for

Aerobic and anaerobic iron oxidizers together drive denitrification and carbon cycling at marine iron-rich hydrothermal vents

Sean M. McAllister[1,#], Rebecca Vandzura[1], Jessica L. Keffer[2], Shawn W. Polson[3], and Clara S. Chan[1,2]

1: School of Marine Science and Policy, University of Delaware, Newark, Delaware, USA
2: Department of Earth Sciences, University of Delaware, Newark, Delaware, USA
3: Center for Bioinformatics and Computational Biology, University of Delaware, Newark, Delaware, USA

#: Currently at the Cooperative Institute for Climate, Ocean, and Ecosystem Studies, University of Washington, Seattle, Washington, USA / Pacific Marine Environmental Laboratory, National Oceanic and Atmospheric Administration, Seattle, Washington, USA

Address correspondence to Clara S. Chan, cschan@udel.edu.

Document includes:

- **Supplemental Table 2.** *Candidatus Ferristratum* sp. MAG abundance, activity, % GC, quality, and summary statistics.
- **Supplemental Table 4.** Counts showing the relative number of MAG bins with validated CRISPR spacers.
- **Supplemental Table 5.** Survey of polysaccharide-degrading enzymes used to understand heterotrophy in the iron mats.
- **Supplemental Figure 1.** 16S rRNA gene maximum likelihood phylogenetic tree (300 bootstraps) showing the placement of all full-length sequences from the DTB120 phylum (branches purple) in relation to the Desulfobacterota, Gallionellaceae/Zetaproteobacteria (Proteobacteria), Aquificae, and Chloroflexi.
- **Supplemental Figure 2.** Full 16S rRNA gene phylogenetic tree showing the placement of DTB120.
- **Supplemental Figure 3.** Heatmaps showing the percent amino acid identity (AAI) for pairwise MAG comparison for all Desulfobacterota and DTB120 in this study. A) continuous color heat ramp. B) discrete color heat ramp.
- **Supplemental Figure 4.** Consensus taxonomy of viral contigs identified by VirSorter.

Supplemental Table 2. *Candidatus Ferristratum* sp. MAG abundance, activity, % GC, quality, and summary statistics.

Name[a]	Rel. Abund. (%)	Total expression (TPM)[b]	% GC	16S present	% Complete	% Redundant	Genome Size (Mb)	No. contigs	N50 length (Kb)
S1_Delta1	1.5	4,184	51.2	X	25.4	0	0.78	158	5.5
S6_Bacteria1*	3.3	23,475	56.1	X	92.3	2.6	2.31	90	52.6
S6_Delta1*	5.4	22,516	50.7	X	93.6	3.6	2.14	103	33.3
S6_Delta2*	5.2	17,183	54.4		92.9	1.3	2.24	72	51.4
S6_Delta3*	1.6	5,990	53.8		91.0	11.9	2.35	194	21.7
S6_Delta4*	1.2	6,845	48.7		93.6	3.0	2.11	121	23.6
S6_Delta5	0.8	1,065	49.7		19.7	0	1.18	347	3.5
S6_Delta6	0.7	457	50.0		9.1	0	0.24	78	3.2
S6_Delta9	0.6	979	58.4		67.4	2.4	1.52	393	4.1
S6_Delta10	0.6	1,439	51.2		19.9	1.3	0.80	222	3.8
S6_Desulfuromonadales1	0.5	186	51.5		9.1	0	0.27	88	3.1
S19_Bacteria6	1.0	5,525	52.4		43.2	0	1.00	232	4.9
S19_Delta1*	6.2	31,742	53.7		91.0	1.0	2.08	51	90.5
S19_Delta2*	3.8	19,732	51.9		77.4	10.2	2.00	217	13.1
S19_Delta3*	1.4	12,297	51.4	X	94.2	18.8	2.58	150	35.4
S19_Delta6*	0.5	828	54.7		70.7	1.4	1.54	313	5.4
S19_Desulfuromonadales1	0.3	2,948	50.6		30.3	1.9	0.86	194	5.0
S19_Desulfuromonadales2	0.6	748	50.1		26.0	1.7	0.45	131	3.4
S19_Desulfuromonadales3	0.5	631	48.1		19.2	0.9	0.48	151	3.1

[a]Bin names with an asterisk were used for metabolic predictions .

[b]S6 totals expression number represent the pre Fe(II) addition time point only.

Supplemental Table 4. Counts showing the relative number of MAG bins with validated CRISPR spacers

Sample	Phyla	Total no. of bins	No. of bins with CRISPR spacers
S1	Zetaproteobacteria	9	5
	Gammaproteobacteria	2	2
S19	Zetaproteobacteria	28	4
	Chloroflexi	3	2
	Desulfobacterota	6	2
	Candidatus Ferristratum sp.	6	0
	Euryarchaeota	1	1
	Methylococcales	3	1
	Myxococcales	3	2
	Planctomycetes	15	4
	Thermodesulfovibrio	1	1
S6	Zetaproteobacteria	12	9
	Desulfobacterota	6	2
	Candidatus Ferristratum sp.	5	2
	Marinimicrobia	4	1

Supplemental Table 5. Survey of polysaccharide-degrading enzymes used to understand heterotrophy in the Fe mats.

	No. bins	Cellulase	Hemicellulase	Endohemicellulases	Other Oligosaccharide Degrading Enzymes	Amyloytic Enzymes	Chitin Degrading Enzymes
S1 – binned	11	5	-	2	1	2	9
S19 – binned	56	13	20	7	23	24	32
S6 – binned	32	6	-	2	6	12	24
S1 – unbinned		13	1	1	17	19	27
S19 – unbinned		82	75	26	100	102	110
S6 – unbinned		43	34	10	29	46	65
S1 – Zetaproteobacteria	5	-	-	-	-	-	5
S6 – Zetaproteobacteria	15	-	-	-	-	-	15
S19 – Zetaproteobacteria	7	-	-	-	-	-	8
S1 – Caldithrix	2	2	-	2	1	2	2
S6 – Chloroflexi	1	3	-	-	1	1	-
S19 – Chloroflexi	2	2	1	-	1	1	3
S19 – Planctomycetes	9	2	11	5	10	7	3
S19 – Marinimicrobia	4	2	-	-	-	4	1
Candidatus Ferristratum sp. – S1_Delta1		-	-	-	-	-	-
Candidatus Ferristratum sp. – S19_Delta1		-	-	-	2	-	-
Candidatus Ferristratum sp. – S19_Delta2		1	-	-	2	-	-
Candidatus Ferristratum sp. – S19_Delta3		-	-	-	-	-	-
Candidatus Ferristratum sp. – S19_Delta6		-	-	-	-	-	-
Candidatus Ferristratum sp. – S6_Bacteria1		1	-	-	1	-	-
Candidatus Ferristratum sp. – S6_Delta1		-	-	-	-	-	-
Candidatus Ferristratum sp. – S6_Delta2		-	-	1	1	-	-
Candidatus Ferristratum sp. – S6_Delta3		-	-	-	-	-	-
Candidatus Ferristratum sp. – S6_Delta4		-	-	-	1	-	-
Candidatus Ferristratum sp. – S6_Delta9		-	-	-	-	-	-

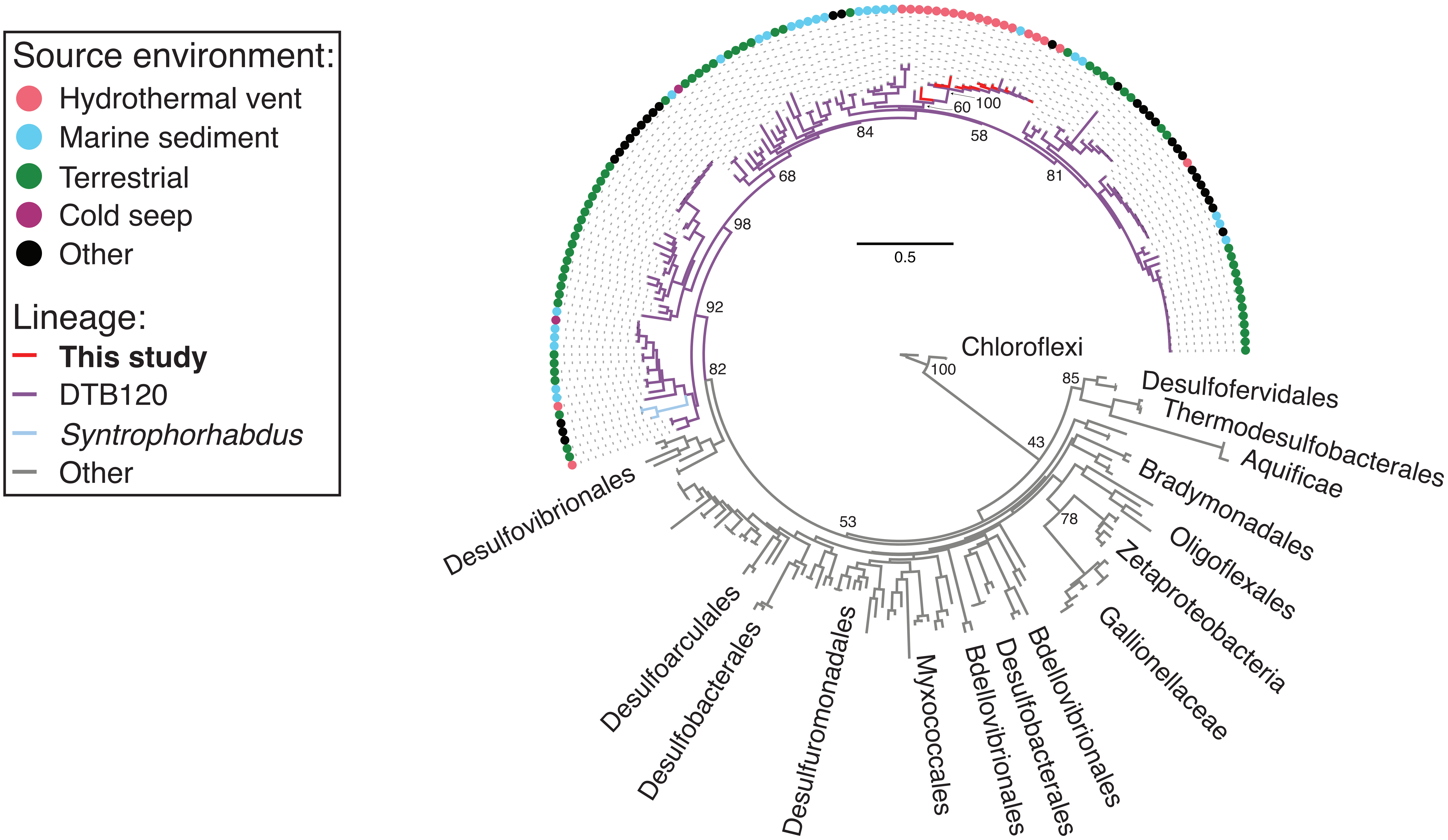

Supplemental Figure 1. 16S rRNA gene maximum likelihood phylogenetic tree (300 bootstraps) showing the placement of all full-length sequences from the DTB120 phylum (branches purple) in relation to the Desulfobacterota, Gallionellaceae/Zetaproteobacteria (Proteobacteria), Aquificae, and Chloroflexi. Sequences from this study (branches red) are distant from the closest isolated representative, *Syntrophorhabdus* sp. (branches blue). Source environments are plotted as dots along the outside of the tree, showing that close relatives to sequences from this study are also primarily found at hydrothermal vents.

Supplemental Figure 2. Full 16S rRNA gene phylogenetic tree showing the placement of DTB120. This figure is an identical tree to Supplemental Figure 1, with each branch labeled.

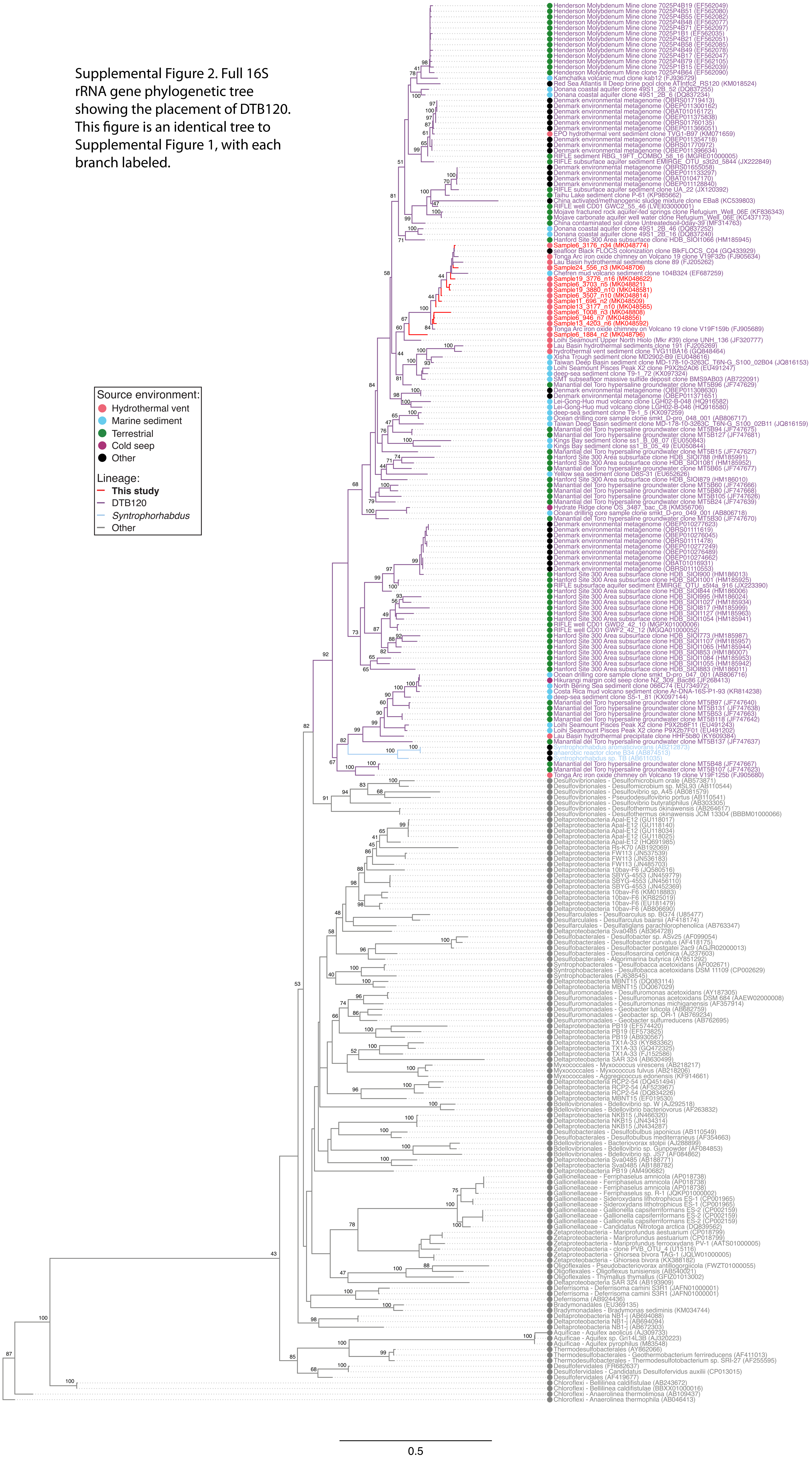

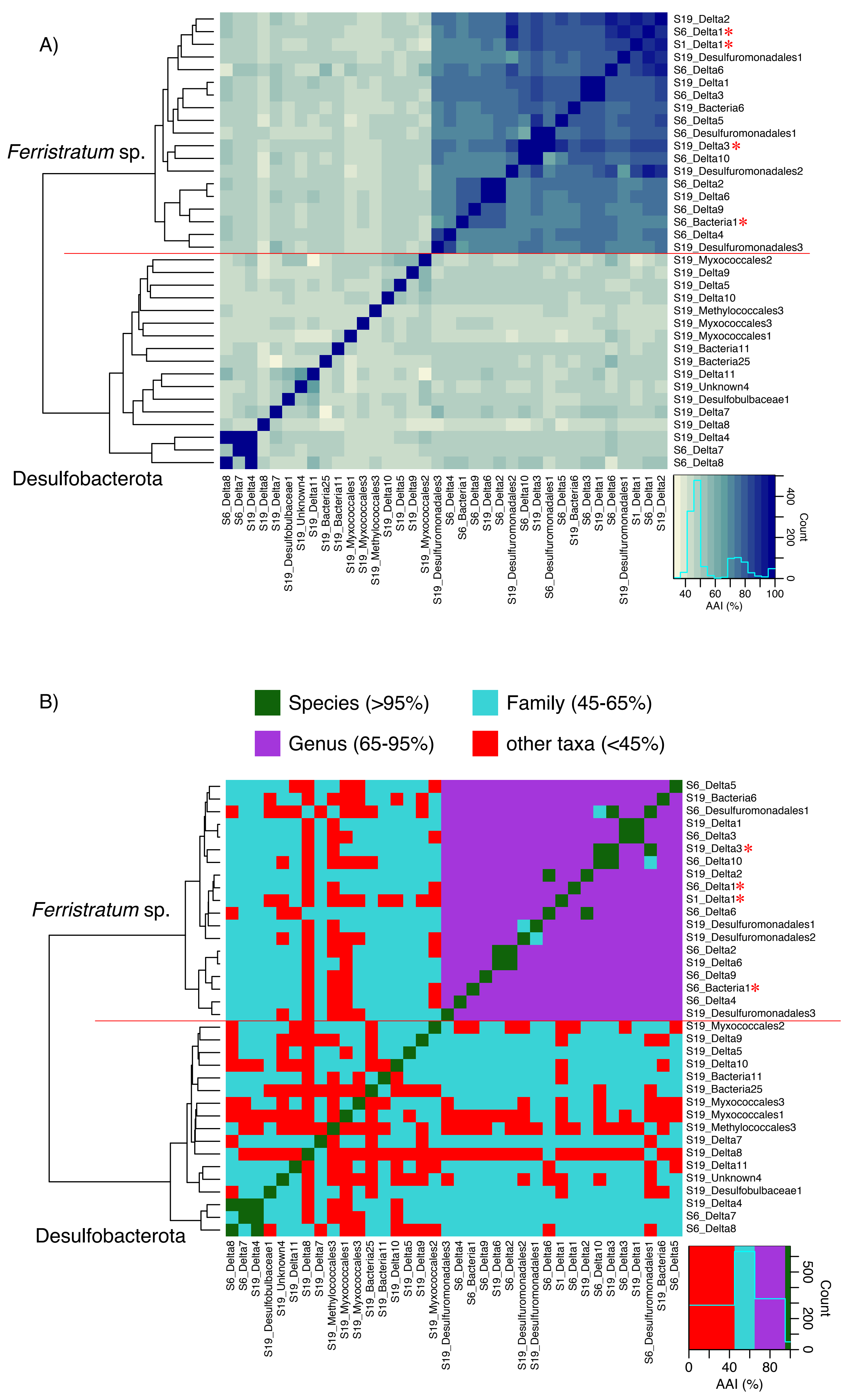

Supplemental Figure 3. Heatmaps showing the percent amino acid identity (AAI) for pairwise MAG comparison for all Desulfobacterota and DTB120 in this study. A) continuous color heat ramp. B) discrete color heat ramp following the guidelines from Konstantinidis et al. (2017) for species, genus, and family level cutoffs.

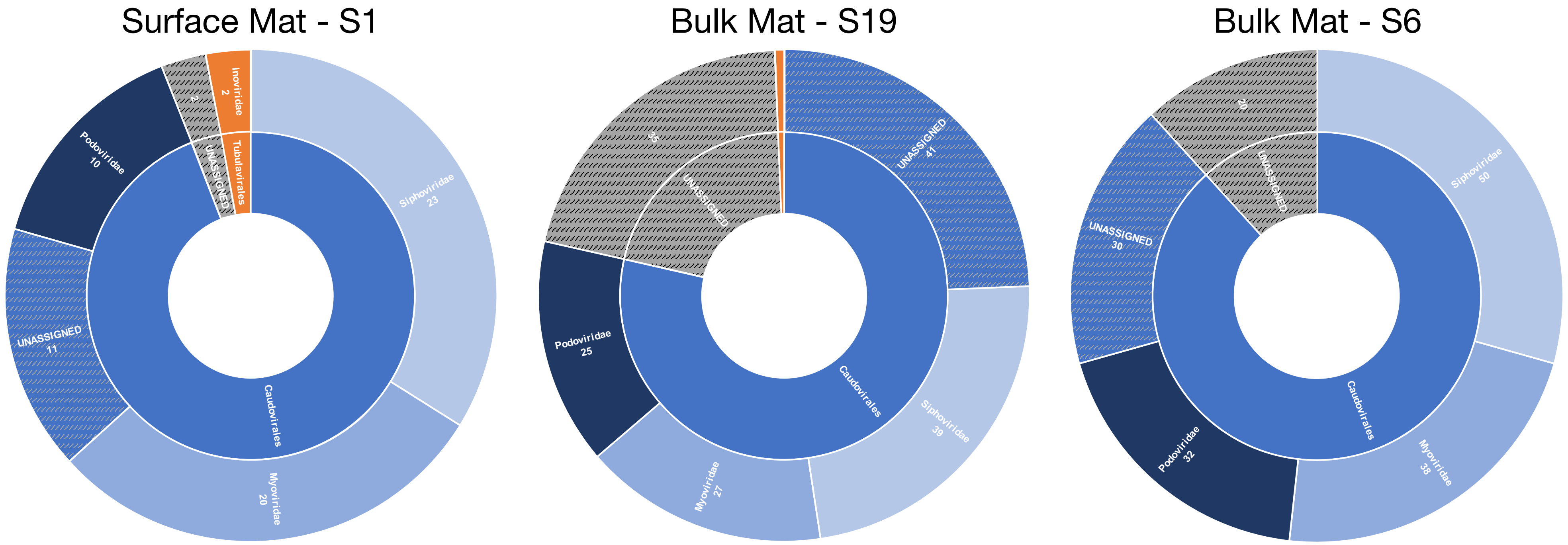

Supplemental Figure 4. Consensus taxonomy of viral contigs identified by VirSorter as category 1/2 (high/medium confidence viral contigs) or 4/5 (high/medium confidence prophage regions). Taxonomy was assigned based on consensus vContact2 cluster taxonomy (priority) or taxonomy of BLASTp hits to Viral RefSeq (consensus of best informative hit per ORF). “UNASSIGNED" includes contigs with no hits and those with hits to viruses with no assigned taxonomy. Inner ring = Order, outer ring = Family.
